# Effectiveness of Ultrasound-Guided Hydrodissection for Atypical Anterior Cutaneous Nerve Entrapment Syndrome Presenting as Lower Back Pain Due to Retrograde Neuropathic Pain

**DOI:** 10.7759/cureus.94871

**Published:** 2025-10-18

**Authors:** Tsuyoshi Yoshinaga, Tetsuya Koga, Hiroaki Koga

**Affiliations:** 1 Department of Low Back Pain and Sacroiliac Joint Rehabilitation, Kagoshima Kyousaikai Nanpuh Hospital, Kagoshima, JPN; 2 Department of Orthopedics, Kagoshima Kyousaikai Nanpuh Hospital, Kagoshima, JPN

**Keywords:** abdominal wall peripheral nerve, anterior cutaneous nerve entrapment syndrome, carnett’s sign, hydrodissection, low back pain, rectus abdominis, retrograde neuropathic pain

## Abstract

This case report describes an atypical presentation of anterior cutaneous nerve entrapment syndrome (ACNES). A male patient with refractory lower back pain without typical abdominal symptoms exhibited localized tenderness in the abdominal wall. The patient’s back pain was reproduced by compression of the tender point. Color Doppler ultrasonography revealed a pulsating perforating artery directly beneath the tender point. Ultrasound-guided hydrodissection was performed at the site, resulting in resolution of Carnett’s sign and significant improvement in both pain and motor function.

This case suggests that retrograde neuropathic pain (RNP) may arise from entrapment of the anterior cutaneous nerve, manifesting as referred pain in the lower back. Even in the absence of abdominal symptoms, a positive Carnett’s sign should prompt the consideration of abdominal wall nerve entrapment and associated RNP in the differential diagnosis of refractory low back pain.

## Introduction

In recent years, the entrapment of peripheral nerves in the abdominal wall has gained attention as an underrecognized cause of low back and pelvic pain [1]. Up to 67% of female patients with chronic pelvic pain report abdominal wall tenderness, which is often attributed to abdominal wall nerves [2]. However, the causal relationship between abdominal wall nerve entrapment and low back pain remains unclear, and reports linking anterior cutaneous nerve entrapment syndrome (ACNES) to low back pain are limited [3].

ACNES is a peripheral neuropathy caused by the entrapment of the anterior cutaneous branches of the intercostal nerves within the abdominal wall, particularly in the rectus sheath [4]. After traversing the internal oblique and transverse abdominis muscles, the intercostal nerves penetrate the anterior rectus sheath to reach the skin. Anatomically dense regions, such as the penetration site and crossing points with perforating arteries, are prone to mechanical irritation and entrapment [5].

Typical symptoms include localized tenderness and sharp pain in the abdominal wall. The male-to-female ratio is approximately 3:7, with pain occurring more frequently on the right side (55%), followed by the left (30%). Bilateral symptoms are least common (13%) [4]. The diagnostic criteria include (1) positive Carnett’s sign (pain exacerbated during abdominal muscle contraction), (2) presence of a tender point within 2 cm medial to the lateral border of the rectus abdominis, (3) detection of pulsating perforating arteries via ultrasound, and (4) pain relief following local anesthetic injection [4,6].

Atypical presentations include visceral-like pain or groin discomfort, which complicate the diagnostic process [4,7].

This report presents a rare case of atypical ACNES without abdominal pain that presented primarily as low back pain. Pain mechanisms, such as retrograde neuropathic pain (RNP) [8] and myofascial pain, may be involved. Ultrasound-guided hydrodissection has been proven effective for both diagnosis and treatment. To aid in understanding these pain mechanisms, Table 1 compares the characteristics of both typical and atypical peripheral nerve entrapment. This report discusses the importance of considering abdominal wall nerve entrapment in refractory low back pain and the therapeutic potential of hydrodissection.

## Case presentation

A 40-year-old male (height, 168 cm; weight, 68 kg) presented to our clinic with a 20-year history of chronic lower back pain, which had recently worsened and was accompanied by radiating pain in both lower limbs. The patient had no history of spinal surgery.

**Figure 1:**
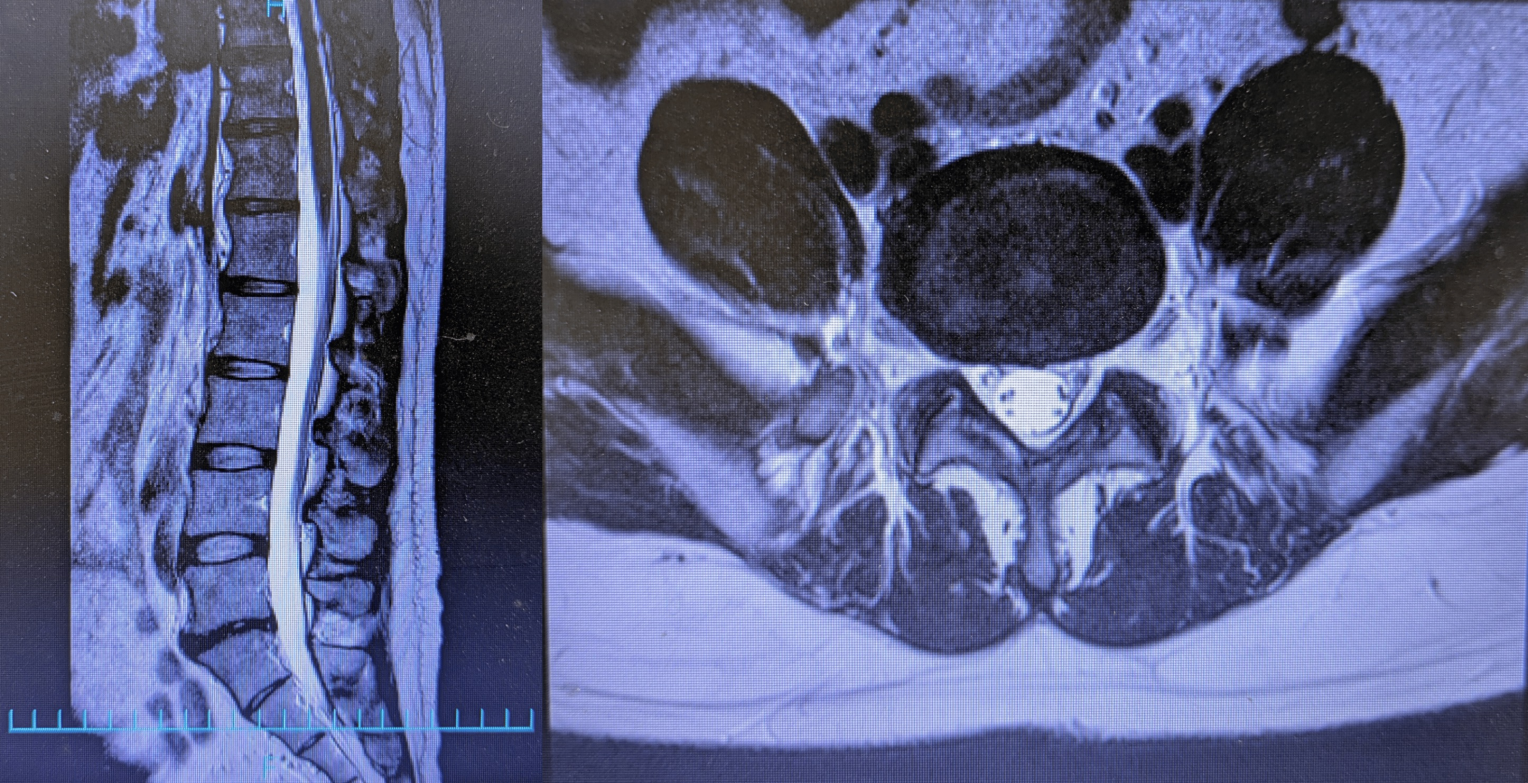
Lumbar MRI showing mild structural changes without evidence of disc herniation

In June 2021, the patient was referred to our institution with worsening lower back pain and newly developed bilateral leg pain. Lumbar magnetic resonance imaging (MRI) revealed only mild structural changes without evidence of disc herniation or neurological deficits (Figure 1). Abdominal and pelvic CT showed no abnormalities. Sacroiliac joint dysfunction was diagnosed based on the physical examination, and conservative treatment, including rehabilitation, was initiated, resulting in temporary symptom relief.

In January 2023, the patient experienced pain recurrence in the lower back and bilateral inguinal regions. The clinical evaluation suggested L5/S1 discogenic pain, and a diagnostic and therapeutic L5/S1 disc block was performed. The procedure led to significant pain relief; however, the discomfort persisted during lumbar extension and right trunk rotation.

Although the patient did not report abdominal pain, a physical examination performed in February 2023 revealed three distinct tender points within 2 cm medial to the lateral border of the right rectus abdominis. These correspond anatomically to the penetration sites of the T9 and T11 intercostal nerves. Compression of these points reproduced the patient’s right lower back pain, which was exacerbated during head elevation (positive Carnett’s sign), suggesting an atypical presentation of ACNES in a male patient, which is typically seen in females with abdominal pain.

Color Doppler ultrasonography of the tender points revealed a pulsating perforating artery directly beneath the site, supporting the diagnosis of ACNES (Video 1). Blood tests and repeat lumbar MRI revealed no abnormalities. ACNES was diagnosed based on the clinical and imaging findings. Laboratory tests, including complete blood count (CBC), liver function (alanine aminotransferase (ALT), aspartate aminotransferase (AST), alkaline phosphatase (ALP), gamma-glutamyl transpeptidase (γ-GTP), lactate dehydrogenase (LDH), total protein), serum amylase, and a basic metabolic panel, were performed to exclude other causes of abdominal wall and lumbar pain, and were all within normal limits. Abdominal ultrasound revealed no significant findings.

**Video 1:** Pulsatile perforating artery detected beneath the tender point To confirm the anatomical correlation between the patient’s tender point and the ultrasonographic finding, firm pressure was reapplied to the site during Doppler ultrasonography (36-43 seconds).

Ultrasound-guided hydrodissection (HD) was subsequently performed to target the penetration sites of the T9-T11 intercostal nerves and adjacent perforating arteries within the right rectus abdominis.

Methods 

Local infiltration anesthesia was administered using 1% carbocaine. The injectate consisted of 10 mL of bicarbonated Ringer’s solution mixed with 0.2 mL of 1% carbocaine per site, totaling 26.5 mL across the three locations. The patient’s allergy history was reviewed prior to administration to confirm the absence of hypersensitivity to local anesthetics. The final concentration of carbocaine (approximately 0.02%) was selected to minimize pharmacological anesthetic effects while prioritizing the mechanical separation effect of HD. This approach aligns with Japanese National Health Insurance regulations, which require a minimal anesthetic dosage for nerve block reimbursement [9].

Bicarbonated Ringer’s solution was chosen over saline because of its near-neutral pH (7.0-8.5) and electrolyte composition, which is similar to that of extracellular fluid. Accordingly, it reduces tissue irritation and improves patient comfort during injection. This rationale is consistent with the theoretical framework proposed by Lam et al. [10].

Using a 23-G, 150 mm PTCD needle under a 9-2 MHz linear probe with a fixed needle guide, an in-plane approach at an angle of 19° was employed. The total procedure time was 65 min.

**Figure 2:**
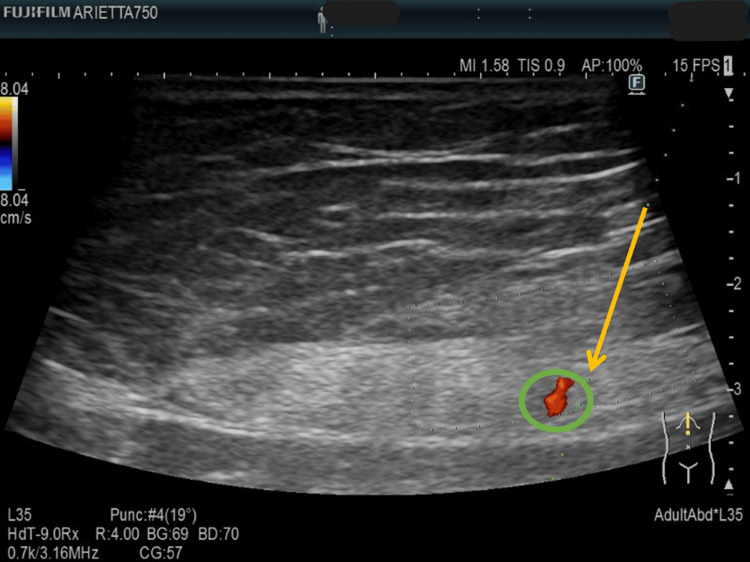
Ultrasound-guided hydrodissection targeting the anterior branch of the intercostal nerve at the point of maximal tenderness The needle insertion path (yellow arrow) was directed toward the perforating artery (green circle) at its pulsation point, targeting the anterior branch of the intercostal nerve penetration site located directly beneath the point of maximal tenderness with a positive Carnett’s sign.

The goal of HD is to mechanically release the anterior cutaneous branches of the intercostal nerves (T9-T11) from the surrounding perimuscular connective tissue (PMCT) and adhesions. Real-time ultrasound guidance was used in the longitudinal view, with the perforating artery serving as a landmark (confirmed using color Doppler in Video 1) [11]. Fluid was injected at the penetration site and formed a longitudinal hypoechoic halo around the vessel, indicating successful separation (Figure 2, Video 2). To minimize vascular injury, small volumes were injected layer by layer from the subcutaneous tissue to the anterior rectus sheath with continuous needle tip visualization.

**Video 2:** Real-time ultrasound-guided hydrodissection procedure Demonstration of the hydrodissection technique for the rectus abdominis muscle

The total volume of injectate (10 mL bicarbonated Ringer’s + 0.2 mL carbocaine per site) was based on literature recommending 5-10 mL per site for effective HD in peripheral nerve entrapment [10]. The volumes were adjusted in real time using ultrasound to optimize tissue separation while avoiding complications.

Needle placement and fluid spread were confirmed using ultrasonography (Figure 2). Video 2 shows the HD technique used in this study.

Pain was assessed using a numerical rating scale (NRS), and functional status was evaluated using the Oswestry Disability Index (ODI). Motor functions, including active straight leg raise (ASLR), gait, and single-leg stance, were recorded by a physical therapist via video observation.

Results

The patient experienced complete resolution of abdominal and low back pain immediately following ultrasound-guided hydrodissection. Carnett’s sign became negative after the procedure. For consistency with the functional assessment, pain scores were recorded using an NRS on the same days as the ODI evaluations.

**Figure 3:**
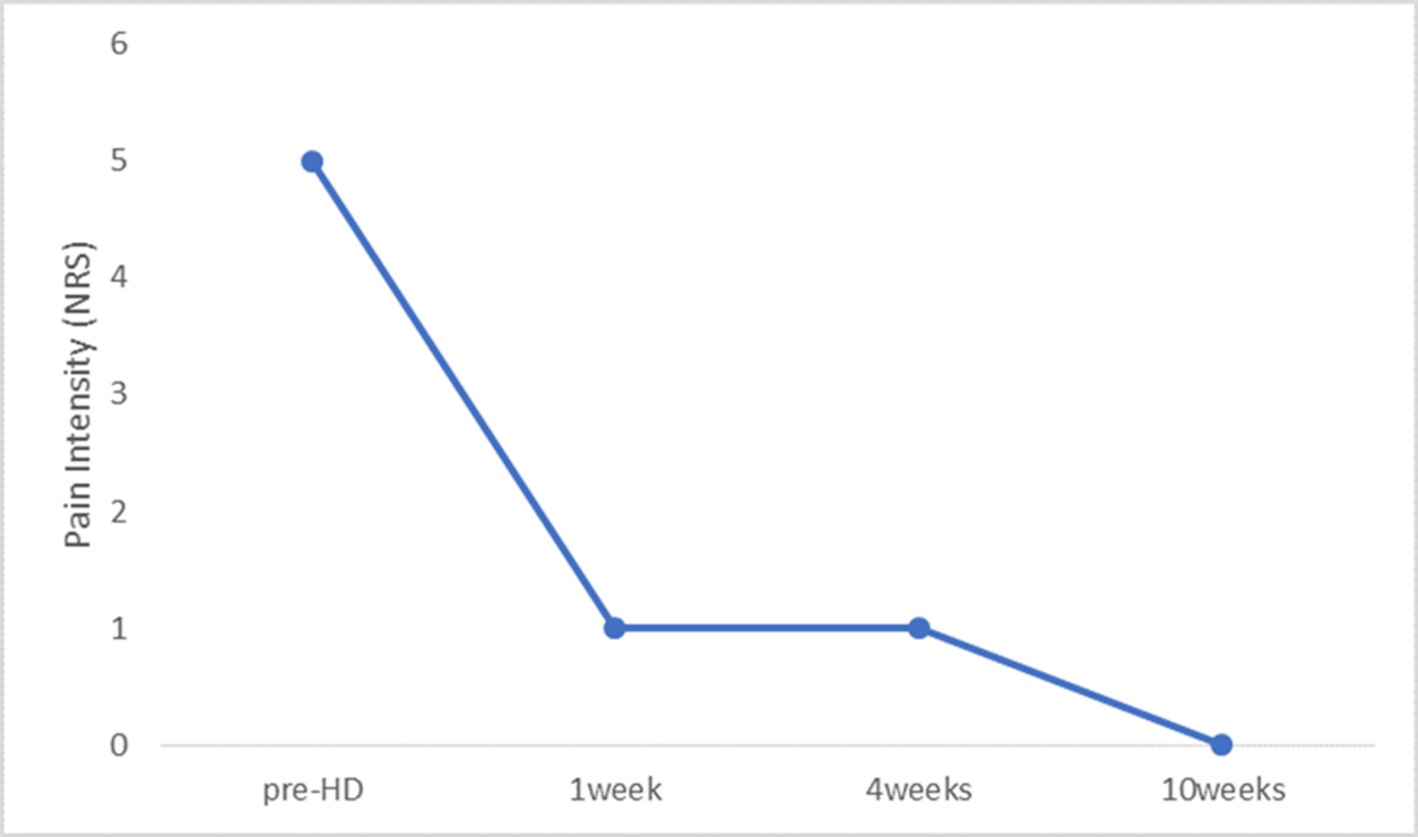
Temporal changes in low back pain severity using the numerical rating scale (NRS) HD, hydrodissection; NRS, numerical rating scale

**Figure 4:**
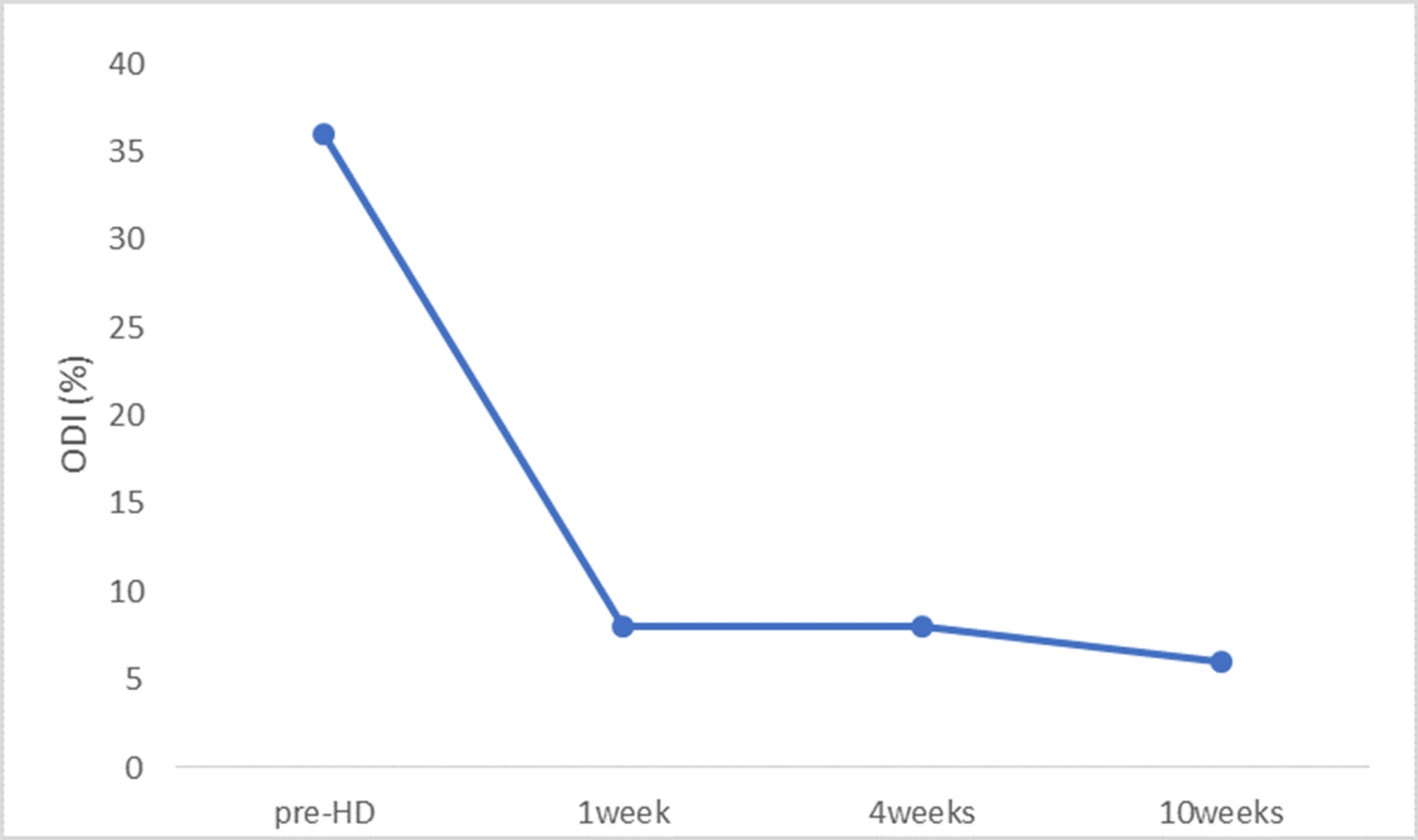
Functional improvement measured by the Oswestry Disability Index (ODI) HD, hydrodissection; ODI, Oswestry Disability Index

The tender points were no longer detectable after treatment, and the patient exhibited marked improvement in both low back pain and motor function. The NRS score improved from 5/10 before treatment to 1/10 at both one and four weeks, and to 0/10 at ten weeks (Figure 3). The ODI score improved from 36% (18/50) to 8% (4/50) at one and four weeks, and to 6% (3/50) at ten weeks (Figure 4).

**Figure 5:**
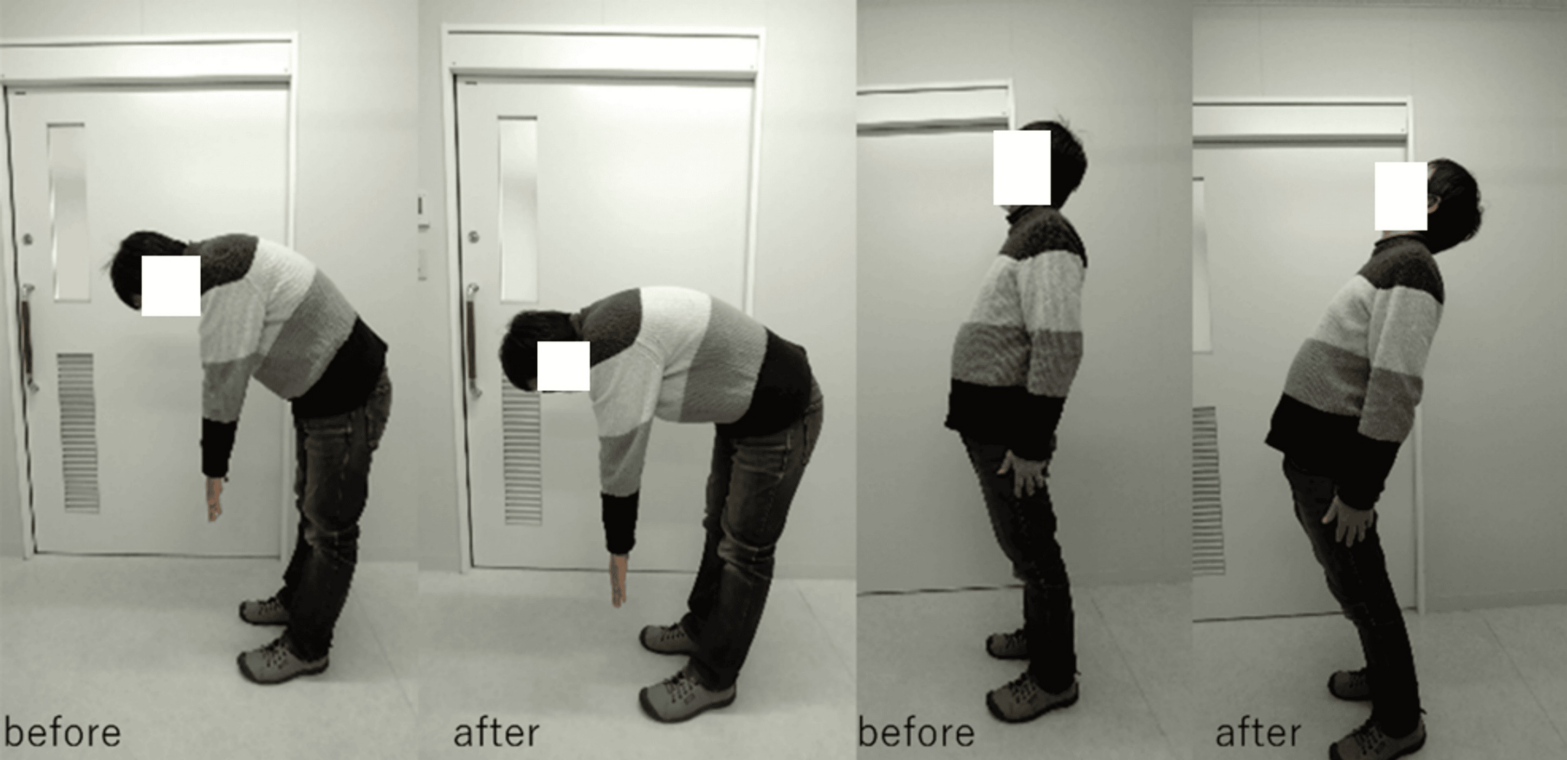
Immediate changes in spinal flexion and extension range of motion before and after hydrodissection This figure illustrates the changes in spinal flexion and extension range of motion observed before and immediately after the hydrodissection (HD) procedure.

Motor function improvements, including trunk flexion/extension, active straight leg raise (ASLR), single-leg stance, and gait, were confirmed through video documentation and physical therapist observations (Figure 5, Video 3). These changes are summarized in Table 1.

**Video 3:** Motor function before and after hydrodissection: immediate functional changes This video demonstrates the functional recovery observed immediately after hydrodissection (HD). Improvements were documented in multiple motor tasks, including forward and backward trunk flexion, active straight leg raise (ASLR), single-leg standing, gait, and stair negotiation. These changes reflect enhanced mobility and neuromuscular control following the procedure.

**Table 1:** Transition of NRS, ODI, and motor function assessed by video evaluation ASLR, active straight leg raise; NRS, numerical rating scale; ODI, Oswestry Disability Index

Time Point	NRS	ODI	Motor Function
Pre-treatment	5/10	36%	Unstable single-leg stance, limited trunk motion, and restricted ASLR
1 week	1/10	8%	Improved
4 weeks	1/10	8%	Improvement maintained
10 weeks	0/10	6%	Improvement maintained

No symptom recurrence was observed during follow-up. Abdominal tenderness and reproducible low back pain completely resolved at four months post-treatment. The patient reported mild discomfort in the lower back after prolonged static sitting. Based on these findings, treatment was considered successful.

A chronological summary of the clinical course and interventions is presented in Table 2.

**Table 2:** Treatment timeline and clinical course in the present case ACNES, anterior cutaneous nerve entrapment syndrome

Period	Clinical Course	Diagnosis / Treatment	Remarks
~20 years ago	Onset of low back pain	—	Cause unknown
Jun-21	Exacerbation of low back pain, onset of bilateral leg pain. Tenderness in the right upper abdomen confirmed.	Diagnosed with sacroiliac joint dysfunction. Conservative treatment initiated.	Temporary symptom relief with conservative therapy
June 2021 – Jan 2023	Disappearance of leg pain, reduction of low back pain. Abdominal tenderness persisted.	Rehabilitation (mobilization of spinal facet joints and sacroiliac joints)	—
Jan-23	Recurrence of bilateral inguinal pain and low back pain	Diagnosed with L5/S1 discogenic pain. L5/S1 disc block performed. The patient was prescribed the following medications: • Loxoprofen Sodium 60 mg, orally, three times daily after meals, for 14 days. • Rebamipide 100 mg, orally, three times daily after meals, for 14 days.	Marked improvement after L5/S1 disc block
Feb-23	Residual low back pain during spinal extension and right trunk rotation. Tender points in the right abdomen reproduced right low back pain upon compression.	Positive Carnett’s sign. Conservative treatment continued. ACNES not diagnosed.	—
Feb 2023 – Nov 2024	Repeated relapse and partial remission	Conservative treatment continued	—
Jul-23	—	The patient was prescribed the following medications: • Loxoprofen Sodium 60 mg, orally, three times daily after meals, for 14 days. • Rebamipide 100 mg, orally, three times daily after meals, for 14 days.	—
Nov-24	Color Doppler ultrasound revealed a pulsating perforating artery beneath the tender point.	Diagnosed with ACNES.	—
Dec-24	Pain reduced to NRS 1/10	Hydrodissection (HD) performed	Low back pain and abdominal tenderness improved simultaneously
10 weeks later	Pain disappeared. Only mild discomfort in the lower back after prolonged sitting.	—	—
End of March 2025	Stable condition at follow-up	Treatment completed	—

This case report was conducted in compliance with the 1964 Declaration of Helsinki and its subsequent amendments. Written informed consent was obtained from the patient for the academic use of clinical information and publication of the case report.

## Discussion

This report presents a rare case of low back pain involving coexisting discogenic pain, sacroiliac joint dysfunction, and atypical ACNES. The patient had chronic low back pain for over two decades, with a diagnosis of sacroiliac joint dysfunction in 2021 and L5/S1 discogenic pain in 2023. Although partial symptom relief was achieved through prior interventions, the residual pain was ultimately attributed to ACNES, a diagnosis that required an additional two years to establish. This delay highlights the low clinical awareness of ACNES and its tendency to be overlooked as a potential cause of low back pain.

ACNES is typically observed in female patients presenting with abdominal pain [4]. However, this case involved a male patient without abdominal symptoms who instead exhibited lower back pain, which is a distinctly atypical clinical picture. Notably, Carnett’s sign was positive not only for abdominal tenderness but also for referred pain in the lower back. This finding suggests that abdominal wall nerve entrapment may manifest as retrograde neuropathic pain (RNP) or retrograde referred pain (Valliex phenomenon).

RNP is characterized by abnormal afferent signaling from a peripheral nerve entrapment site toward the central nervous system, resulting in pain being perceived in regions not directly innervated by the affected nerve. In this case, the recurrence of lower back pain upon compression of the abdominal tender point, absence of significant findings on lumbar MRI, and improvement in pain and motor function following hydrodissection all support the involvement of RNP. Similar mechanisms have been reported in other conditions such as shoulder pain secondary to carpal tunnel syndrome and lower back pain associated with ankle instability [8,12]. It is plausible that ACNES triggers RNP through comparable pathways [13]. To aid in understanding these pain mechanisms, Table 3 compares the characteristics of both typical and atypical peripheral nerve entrapment.

**Table 3:** Comparison of typical and atypical peripheral neuropathy features, including retrograde neuropathic pain (RNP) RNP, retrograde neuropathic pain

Feature	Typical Peripheral Neuropathy	Atypical Peripheral Neuropathy (RNP)
Symptom Location	Distal nerve distribution (peripheral)	Proximal to the site of entrapment
Main Symptoms	Numbness, distal pain, muscle weakness	Central pain, restricted range of motion
Imaging Findings	May explain peripheral nerve pathology	No clear abnormalities in proximal structures
Diagnostic Clue	Symptoms match nerve distribution	Proximal symptoms improve after distal intervention

In this case, hydrodissection was performed at the penetration sites of the T9-T11 intercostal nerves within the rectus abdominis using the perforating artery as a landmark. The formation of a fluid halo and immediate resolution of Carnett’s sign indicated successful mechanical decompression. Post-treatment improvements were observed not only in pain scores, but also in motor function, including ASLR and trunk flexion. These results suggest that hydrodissection enhances both neural function and fascial gliding within the perimuscular connective tissue (PMCT) [14].

The injectate contained a highly diluted concentration of carbocaine (final concentration ~0.02%), minimizing the pharmacological anesthetic effects. Although the immediate disappearance of tenderness and Carnett’s sign raises the possibility of an anesthetic influence, the sustained improvement over 10 weeks and consistent reductions in the ODI and NRS scores suggest that mechanical decompression via hydrodissection played a significant therapeutic role.

The coexistence of multiple sources of pain, prior interventions, natural fluctuations in symptoms, and placebo effects are potential confounding factors. However, the temporal association between hydrodissection and symptom resolution, reversal of Carnett’s sign, and improvement exceeding the minimal clinically important difference (MCID) for ODI (13-15 points) provides objective support for the efficacy of the intervention [15].

This case highlights the importance of considering ACNES and RNP in the differential diagnosis of image-negative low back pain. This finding highlights the potential of hydrodissection as a minimally invasive and effective therapeutic option. Future studies should aim to quantify PMCT structural changes and gliding properties via ultrasound imaging and investigate the pathophysiology and treatment outcomes of ACNES and RNP in larger patient cohorts.

This case report is subject to the inherent limitations of a single-patient study, including the inability to establish definitive causality and the potential confounding factors arising from multiple sources of pain. The patient had documented sacroiliac joint dysfunction and discogenic pain, with partial symptom relief following prior interventions, namely, conservative therapy in 2019 and an L5/S1 disc block in 2023. Although the temporal relationship between hydrodissection and complete symptom resolution (e.g., NRS improvement from 5/10 to 0/10 at 10 weeks) is compelling, the delayed effects of previous treatments or natural symptom fluctuations cannot be entirely excluded.

The overlapping nature of pain generators in chronic conditions, such as ACNES, may introduce interpretive bias, a diagnostic challenge noted in the literature [5,16]. Additionally, placebo effects and patient expectations may have contributed to symptom improvement. However, objective findings, such as the resolution of Carnett’s sign and functional improvement exceeding the minimal clinically important difference (MCID) for ODI, help mitigate these concerns [15].

Another limitation is the difficulty in distinguishing between the pharmacological effects of the local anesthetic and the mechanical decompression achieved through hydrodissection. In this case, the injectate contained only 0.2 mL of 1% carbocaine per site (final concentration ~0.02%), suggesting minimal anesthetic influence. Nonetheless, the immediate disappearance of tenderness and Carnett’s sign raised the possibility of an anesthetic contribution. However, the sustained improvement observed over 10 weeks supports the hypothesis that mechanical separation plays a significant role. Nevertheless, further studies are needed to isolate these effects. Comparative trials involving hydrodissection versus local anesthetic injection alone may help clarify the independent therapeutic value of fluid-based tissue separation.

Finally, the follow-up period was relatively short (10 weeks), and stability was confirmed at four months. Although early sustained effects have been demonstrated, long-term efficacy and recurrence rates remain unknown. Previous literature on ACNES cases reports recurrence rates of 20-50% in long-term cohorts, underscoring the need for extended follow-up over 6 to 12 months.

## Conclusions

This case highlights the importance of including anterior cutaneous nerve entrapment syndrome (ACNES) and retrograde neuropathic pain (RNP) in the differential diagnosis of chronic low back pain, particularly when imaging studies fail to reveal abnormalities. Although ACNES is typically associated with abdominal pain in female patients, this report describes a rare presentation of ACNES in a male patient without abdominal symptoms, manifesting instead as lower back pain, underscoring the clinical variability and diagnostic challenges associated with this condition.

Ultrasound-guided hydrodissection targeting the penetration sites of the T9-T11 anterior cutaneous branches resulted in marked improvements in pain scores (NRS), functional status (ODI), resolution of Carnett’s sign, and restoration of motor function. These findings suggest that RNP due to nerve entrapment may have contributed to the patient’s symptoms, and that hydrodissection may have improved both neural function and fascial mobility.

## References

[1] Maatman RC, Boelens OB, Scheltinga MR, Roumen RM (2019). Chronic localized back pain due to entrapment of cutaneous branches of posterior rami of the thoracic nerves (POCNES): a case series on diagnosis and management. J Pain Res.

[2] Mui J, Allaire C, Williams C, Yong PJ (2016). Abdominal wall pain in women with chronic pelvic pain. J Obstet Gynaecol Can.

[3] Kakura K, Harada Y, Shimizu T (2023). A combination of three nerve entrapment syndromes, which was difficult to differentiate from a vertebral compression fracture. Eur J Case Rep Intern Med.

[4] Mol FM, Maatman RC, De Joode LE, Van Eerten P, Scheltinga MR, Roumen R (2021). Characteristics of 1116 consecutive patients diagnosed with anterior cutaneous nerve entrapment syndrome (ACNES). Ann Surg.

[5] Koop H, Koprdova S, Schürmann C (2016). Chronic abdominal wall pain. Dtsch Arztebl Int.

[6] Hata J, Imamura H (2020). Sonographic diagnosis of abdominal cutaneous nerve entrapment syndrome: a report of two cases. Jpn J Gastroenterol Hepatol.

[7] Jacobs ML, Scheltinga MR, Roumen RM (2021). Persistent pain relief following a single injection of a local anesthetic for neuropathic abdominal wall and groin pain. Scand J Pain.

[8] Hagiwara Y, Nakamura T, Sonoki K, Moroi K, Morimoto S, Natsume Y, Yoshida R (2022). "Idiopathic" shoulder pain and dysfunction from carpal tunnel syndrome and cubital tunnel syndrome. Plast Reconstr Surg Glob Open.

[9] (2025). Ministry of Health, Labour and Welfare (MHLW). Diagnostic and therapeutic procedures covered under national health insurance. https://www.mhlw.go.jp/stf/seisakunitsuite/bunya/kenkou_iryou/iryouhoken/iryouhoken15/index.html.

[10] Lam KH, Hung CY, Chiang YP, Onishi K, Su DC, Clark TB, Reeves KD (2020). Ultrasound-guided nerve hydrodissection for pain management: rationale, methods, current literature, and theoretical mechanisms. J Pain Res.

[11] Watari T, Otsuka Y, Shiraishi Y (2025). Precision trigger point injections: an anatomically guided approach for anterior cutaneous nerve entrapment syndrome treatment. Intern Med.

[12] Hagiwara Y, Natsume Y, Wagatsuma T, Hasegawa T, Yoshida R (2024). Low back pain caused by traction peripheral neuropathy due to chronic ankle instability: three clinical cases. Cureus.

[13] Sheila Clarke, Saravanakumar Kanakarajan (2015). Abdominal cutaneous nerve entrapment syndrome. Continuing Education in Anaesthesia Critical Care & Pain.

[14] Larivière C, Preuss R, Gagnon DH, Mecheri H, Driscoll M, Henry SM (2023). The relationship between clinical examination measures and ultrasound measures of fascia thickness surrounding trunk muscles or lumbar multifidus fatty infiltrations: an exploratory study. J Anat.

[15] Copay AG, Glassman SD, Subach BR, Berven S, Schuler TC, Carreon LY (2008). Minimum clinically important difference in lumbar spine surgery patients: a choice of methods using the Oswestry Disability Index, Medical Outcomes Study questionnaire Short Form 36, and pain scales. Spine J.

[16] Boelens OB, Scheltinga MR, Houterman S, Roumen RM (2011). Management of anterior cutaneous nerve entrapment syndrome in a cohort of 139 patients. Ann Surg.

